# Supplementary material for: Discovery of potential targets of Triptolide through inverse docking in ovarian cancer cells
Source: PeerJ. 2020 Mar 18;8:e8620. doi: 10.7717/peerj.8620 (PMC7085293; doi:10.7717/peerj.8620)
Supplement: Supplemental Information 1 [file peerj-08-8620-s001.docx]

An investigation of novel targets in ovarian cancer cells treatment with Triptolide

Wu Qinhang^1^,^*^, Bao Gang^1^, Pan Yang^1^, Qian Xiaoqi^1^, Gao Furong^1^

^1^ Department of Medicinal Chemistry, Nanjing University of Chinese Medicine, Nanjing 210038, China

* Corresponding authors: Address: Nanjing University of Chinese Medicine, Nanjing 210038, China

Tel/Fax: +86 025 85811916. E-mail address: wuqinhang@163.com

Address correspondence to: E-mail: [wuqinhang@163.com](mailto:wuqinhang@163.com)

1. Effects of Cisplatin on the growth of endometrial and SKOV-3 cell lines in vitro.


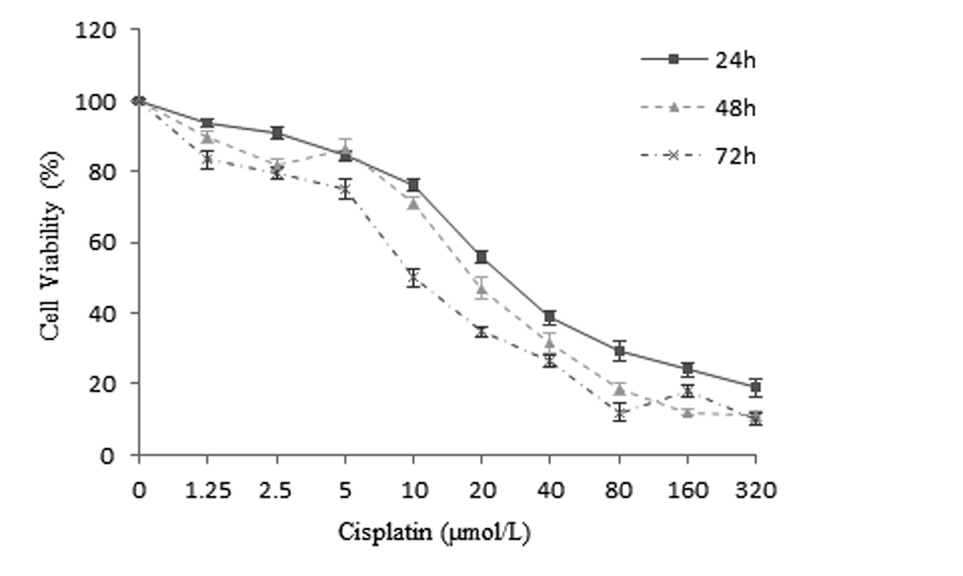


Figure S1. Effects of Cisplatin on the growth of endometrial and SKOV-3 cell lines in vitro.
