# Supplementary material for: Discovery of potential targets of Triptolide through inverse docking in ovarian cancer cells
Source: PeerJ. 2020 Mar 18;8:e8620. doi: 10.7717/peerj.8620 (PMC7085293; doi:10.7717/peerj.8620)
Supplement: Supplemental Information 2 [file peerj-08-8620-s002.zip › Data for mitochondrial membrane potential/images and results for mitochondrial membrane potential.docx]

positive

negative

10NM

20NM

| **Group** | **green fluorescence(%)** |
| --- | --- |
| positive | 30.72 |
| nagetive | 13.20 |
| 10nM | 26.25 |
| 20nM | 40.73 |
