# Supplementary material for: Discovery of potential targets of Triptolide through inverse docking in ovarian cancer cells
Source: PeerJ. 2020 Mar 18;8:e8620. doi: 10.7717/peerj.8620 (PMC7085293; doi:10.7717/peerj.8620)
Supplement: Supplemental Information 7 [file peerj-08-8620-s007.zip › fig4_processing data of mass spectrometry_part3/L3/L3.docx]

**Peptide View**

MS/MS Fragmentation of **LLPDNAILHANSSIVFEGESILEK**
Found in **gi|498331028** in **NCBInr**, microcin C ABC transporter ATP-binding protein [Vibrio campbellii]

Match to Query 3: 2608.536224 from(2609.543500,1+) intensity(3613.4471) index(2)
Data file DATA.TXT
