# Supplementary material for: Discovery of potential targets of Triptolide through inverse docking in ovarian cancer cells
Source: PeerJ. 2020 Mar 18;8:e8620. doi: 10.7717/peerj.8620 (PMC7085293; doi:10.7717/peerj.8620)
Supplement: Supplemental Information 7 [file peerj-08-8620-s007.zip › fig4_processing data of mass spectrometry_part3/L4/L4.docx]

**Peptide View**

MS/MS Fragmentation of **VGGNTTASKADAEDELALGLR**
Found in **gi|297242391** in **NCBInr**, outer membrane protein U [Vibrio parahaemolyticus]

Match to Query 2: 2087.040324 from(2088.047600,1+) intensity(583.9341) index(1)
Data file DATA.TXT
