# Supplementary figures and images for: Discovery of potential targets of Triptolide through inverse docking in ovarian cancer cells
Source: PeerJ. 2020 Mar 18;8:e8620. doi: 10.7717/peerj.8620 (PMC7085293; doi:10.7717/peerj.8620)

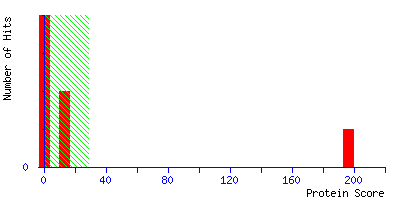

Supplement: Supplemental Information 7 [file peerj-08-8620-s007.zip › fig4_processing data of mass spectrometry_part3/A/score_gif.gif]

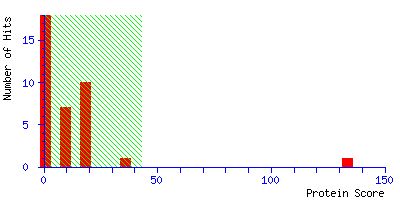

Supplement: Supplemental Information 7 [file peerj-08-8620-s007.zip › fig4_processing data of mass spectrometry_part3/B/score_gif.gif]

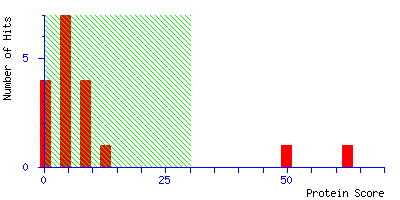

Supplement: Supplemental Information 7 [file peerj-08-8620-s007.zip › fig4_processing data of mass spectrometry_part3/C/score_gif.gif]

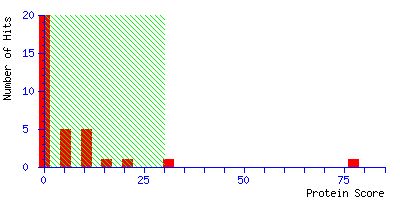

Supplement: Supplemental Information 7 [file peerj-08-8620-s007.zip › fig4_processing data of mass spectrometry_part3/s1/score_gif.gif]

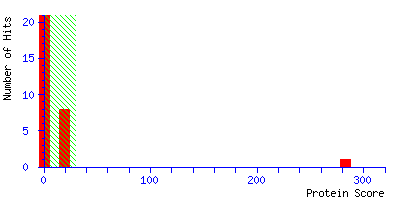

Supplement: Supplemental Information 7 [file peerj-08-8620-s007.zip › fig4_processing data of mass spectrometry_part3/s10/score_gif.gif]

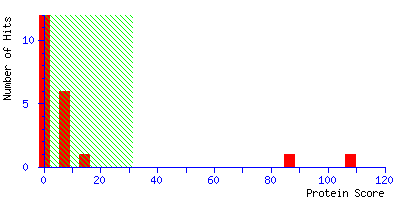

Supplement: Supplemental Information 7 [file peerj-08-8620-s007.zip › fig4_processing data of mass spectrometry_part3/s11/score_gif.gif]

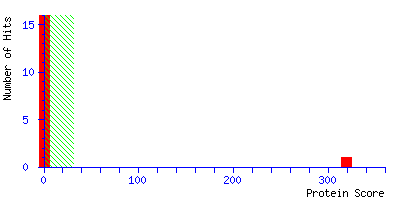

Supplement: Supplemental Information 7 [file peerj-08-8620-s007.zip › fig4_processing data of mass spectrometry_part3/s13/score_gif.gif]

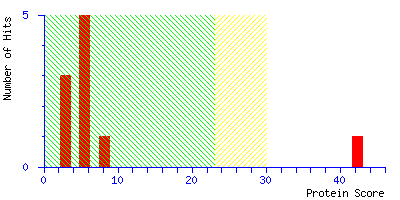

Supplement: Supplemental Information 7 [file peerj-08-8620-s007.zip › fig4_processing data of mass spectrometry_part3/s2/score_gif.gif]

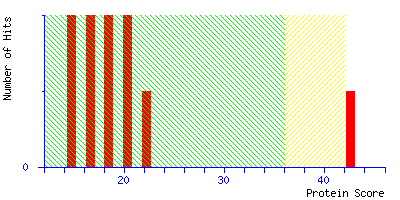

Supplement: Supplemental Information 7 [file peerj-08-8620-s007.zip › fig4_processing data of mass spectrometry_part3/s3/score_gif.gif]

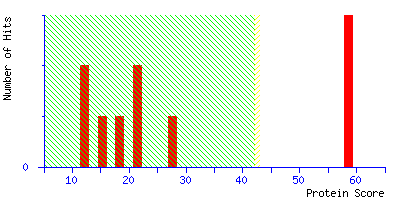

Supplement: Supplemental Information 7 [file peerj-08-8620-s007.zip › fig4_processing data of mass spectrometry_part3/s5/score_gif.gif]

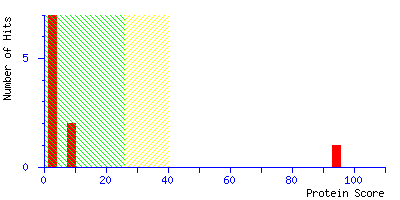

Supplement: Supplemental Information 7 [file peerj-08-8620-s007.zip › fig4_processing data of mass spectrometry_part3/s6/score_gif.gif]

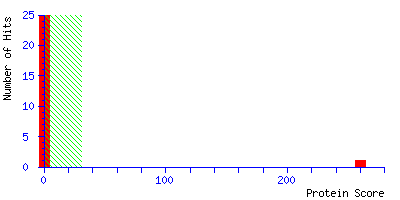

Supplement: Supplemental Information 7 [file peerj-08-8620-s007.zip › fig4_processing data of mass spectrometry_part3/s9/score_gif.gif]

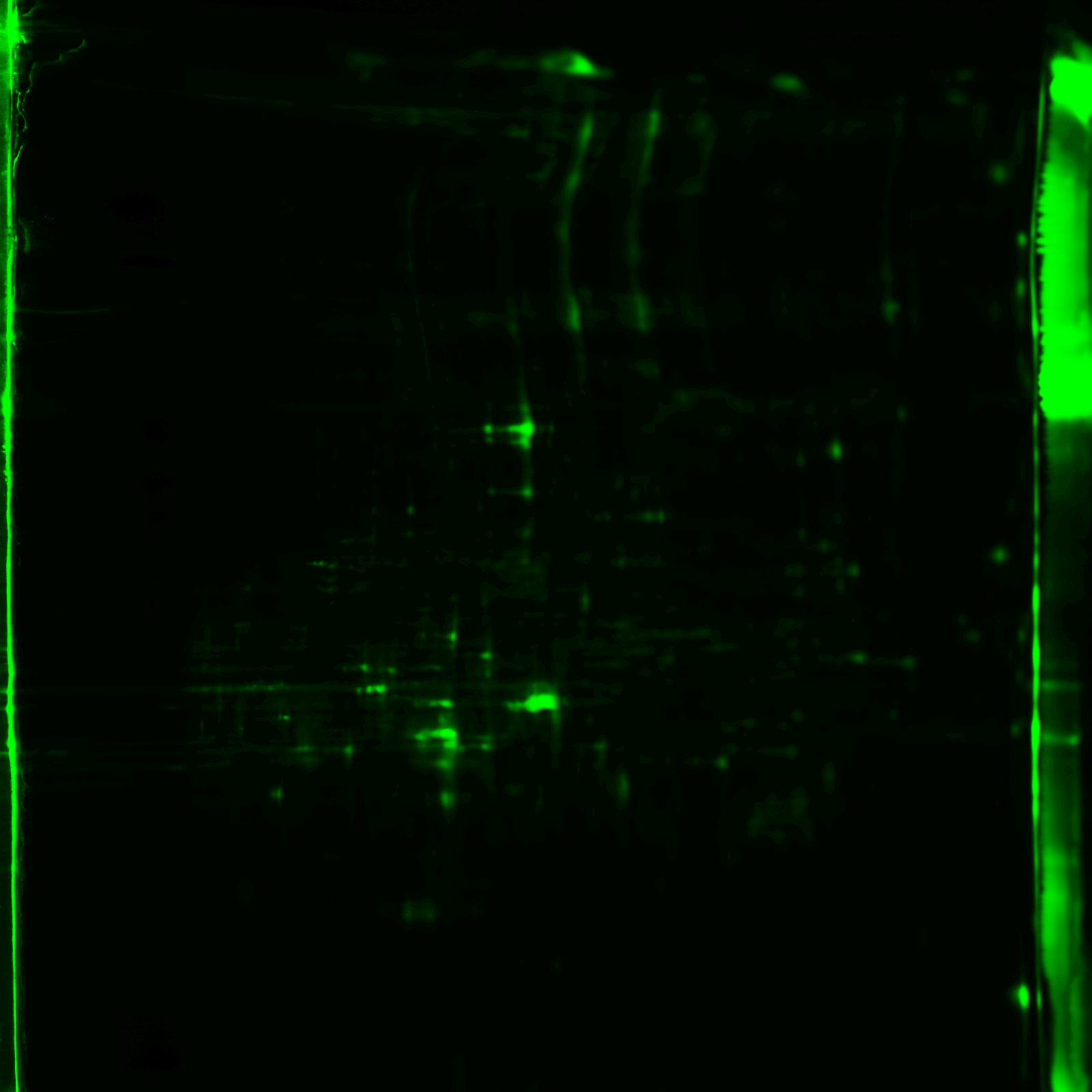

Supplement: Supplemental Information 9 [file peerj-08-8620-s009.zip › cy5.png]

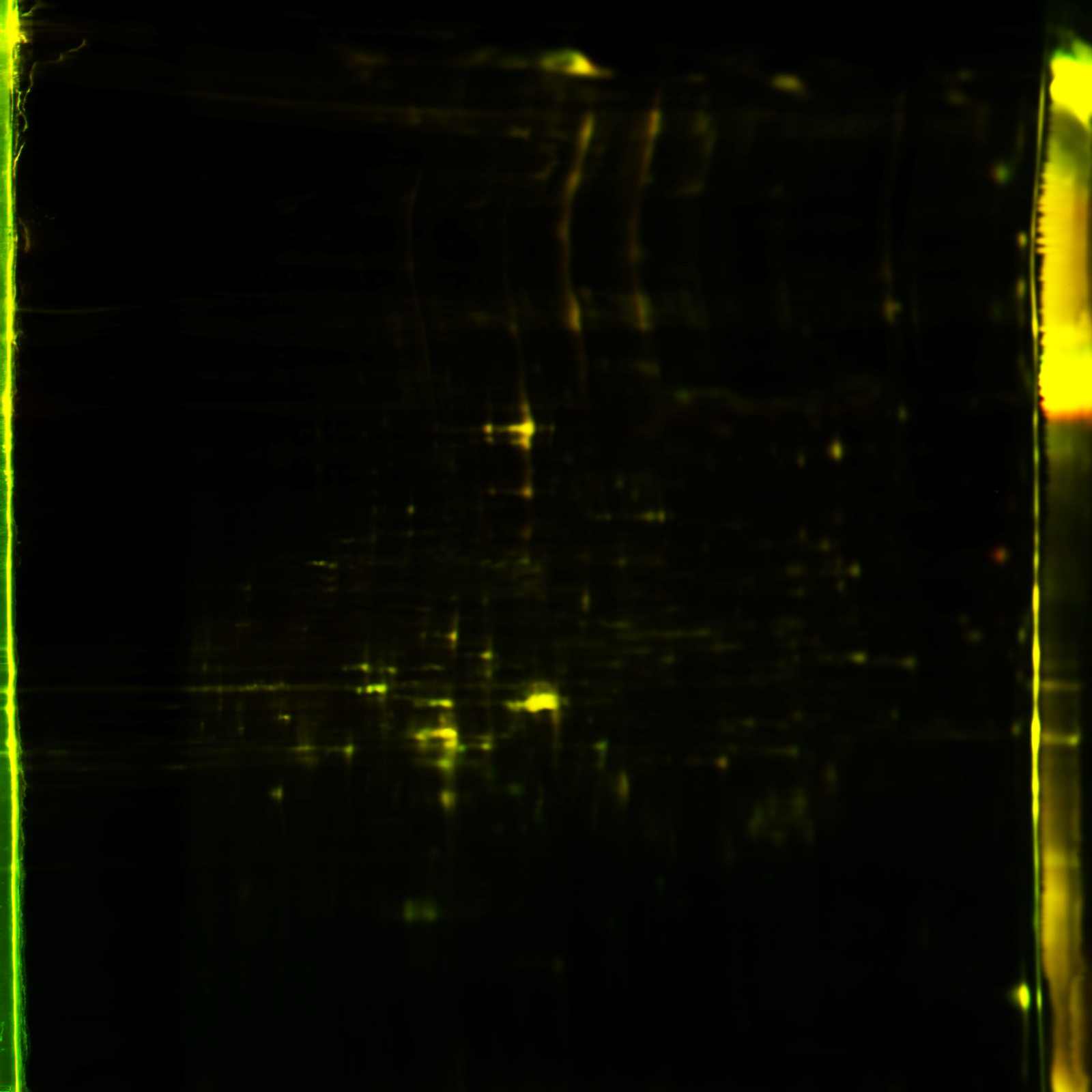

Supplement: Supplemental Information 9 [file peerj-08-8620-s009.zip › overlap.png]

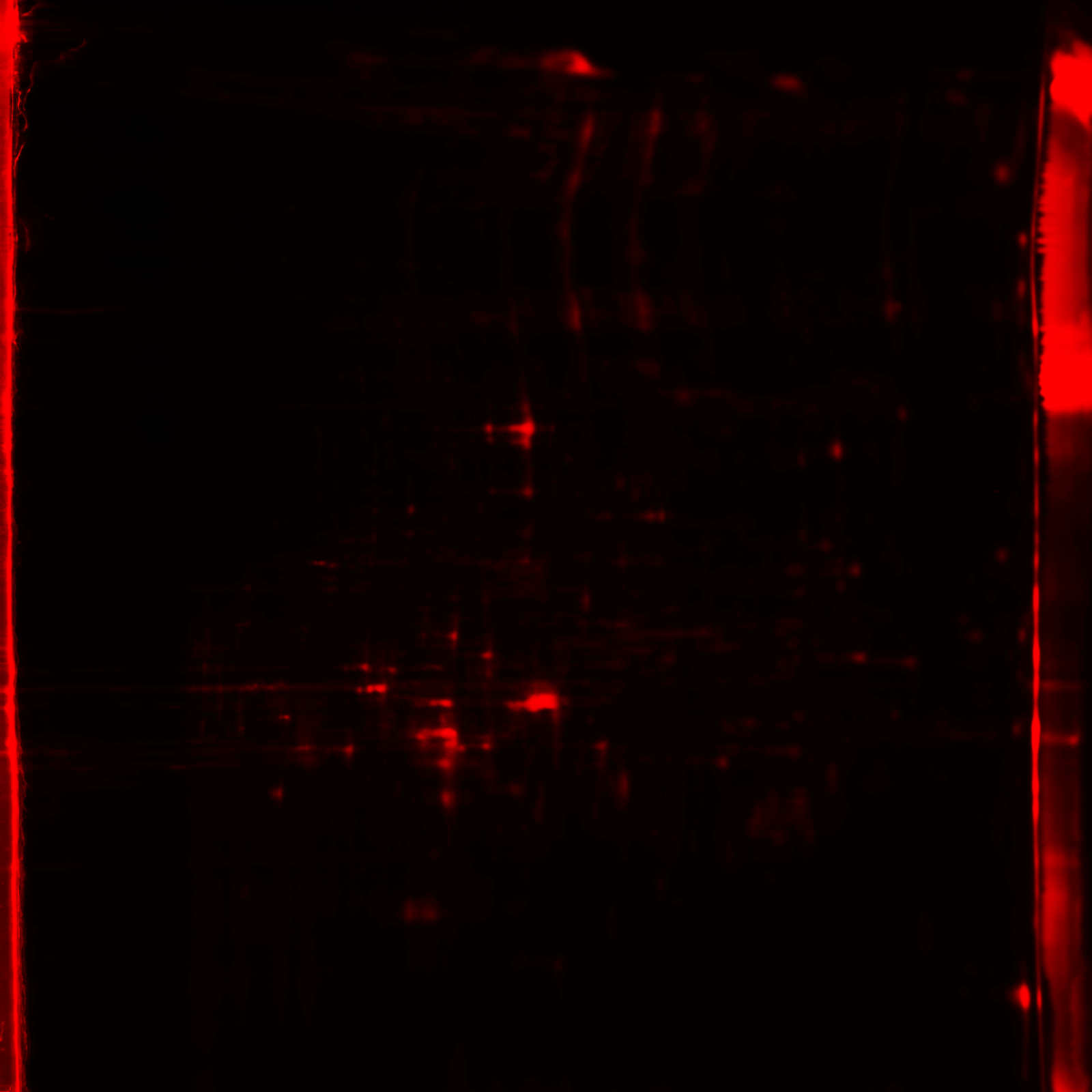

Supplement: Supplemental Information 9 [file peerj-08-8620-s009.zip › cy3.png]

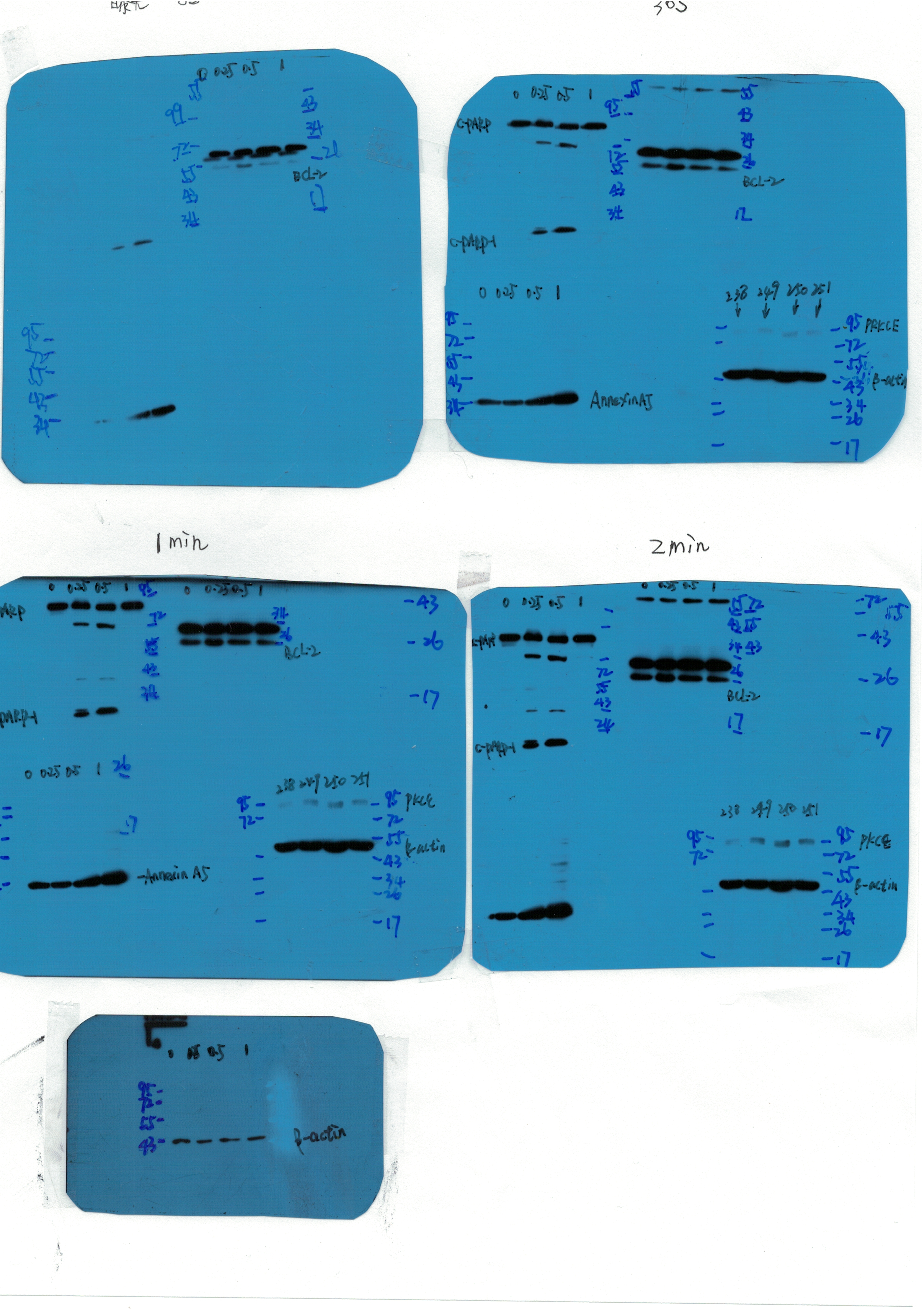

Supplement: Supplemental Information 10 — (A) Validation of differential expression proteins by Western Blotting. (B) The histogram of the relative protein expression levels (equates to protein/β-actin) [file peerj-08-8620-s010.zip › the full-length uncropped blots of figure 5.tif]
