# Supplementary material for: Discovery of potential targets of Triptolide through inverse docking in ovarian cancer cells
Source: PeerJ. 2020 Mar 18;8:e8620. doi: 10.7717/peerj.8620 (PMC7085293; doi:10.7717/peerj.8620)
Supplement: Supplemental Information 8 [file peerj-08-8620-s008.docx]

2

the primary mass spectrometry

the secondary mass spectrometry

3

the primary mass spectrometry

the secondary mass spectrometry

A

the primary mass spectrometry

the secondary mass spectrometry

B

the primary mass spectrometry

the secondary mass spectrometry

C

the primary mass spectrometry

the secondary mass spectrometry

C1

the primary mass spectrometry

the secondary mass spectrometry

Cp

the primary mass spectrometry

the secondary mass spectrometry

d1

the primary mass spectrometry

the secondary mass spectrometry

、

d11

the primary mass spectrometry

the secondary mass spectrometry

d10

the primary mass spectrometry

the secondary mass spectrometry

d13

the primary mass spectrometry

the secondary mass spectrometry

d2

the primary mass spectrometry

the secondary mass spectrometry

d3

the primary mass spectrometry

the secondary mass spectrometry

d4

the primary mass spectrometry

the secondary mass spectrometry

d5

the primary mass spectrometry

the secondary mass spectrometry

d6

the primary mass spectrometry

the secondary mass spectrometry

d7

the primary mass spectrometry

the secondary mass spectrometry

d8

the primary mass spectrometry

the secondary mass spectrometry

d9

the primary mass spectrometry

the secondary mass spectrometry
